# Penta­aqua­(acetonitrile-κ*N*)zinc(II) 4,6-dihydroxy­benzene-1,3-disulfonate trihydrate

**DOI:** 10.1107/S1600536810006525

**Published:** 2010-02-27

**Authors:** Bu-Yun Xie, Wei Huang, Ying Zhang, Rui-Qing Yang, Yong-Rong Xie

**Affiliations:** a Key Laboratory of Jiangxi University for Functional Materials Chemistry, Department of Chemistry and Life Science, Gannan Normal University, Ganzhou, Jiangxi 341000, People’s Republic of China

## Abstract

In the title compound, [Zn(CH_3_CN)(H_2_O)_5_](C_6_H_4_O_8_S_2_)·3H_2_O, the Zn^II^ ion lies on a mirror plane and is octa­hedrally coordinated by one acetonitrile ligand and five water mol­ecules. The 4,6-dihydroxy­benzene-1,3-disulfonate anion, acting as a counter-ion, is also located on the mirror plane. The crystal packing is stabilized by O—H⋯O hydrogen bonds, forming a three-dimensional supra­molecular network.

## Related literature

For general background to the design and construction of coordination compounds of benzene­sulfonic acid derivatives, see: Arnold *et al.* (2001 ▶); Du *et al.* (2006 ▶); Junk & Steed (2007 ▶); Xie *et al.* (2002 ▶); Zhang *et al.* (2009 ▶). For related structures, see: Adarsh *et al.* (2008 ▶); Francis *et al.* (2003 ▶); Lu *et al.* (2008 ▶).
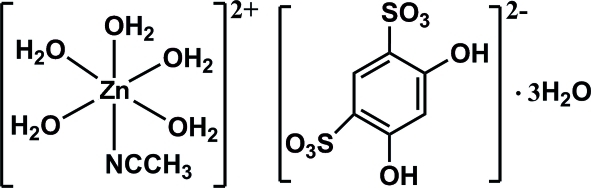


## Experimental

### 

#### Crystal data

[Zn(C_2_H_3_N)(H_2_O)_5_](C_6_H_4_O_8_S_2_)·3H_2_O
*M* *_r_* = 518.80
Orthorhombic, 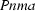

*a* = 12.8731 (10) Å
*b* = 6.9972 (6) Å
*c* = 22.9980 (17) Å
*V* = 2071.6 (3) Å^3^
*Z* = 4
Mo *K*α radiation
μ = 1.46 mm^−1^
*T* = 296 K
0.32 × 0.24 × 0.16 mm

#### Data collection

Rigaku Mercury2 CCD diffractometer
Absorption correction: multi-scan (*CrystalClear*; Rigaku, 2005 ▶) *T*_min_ = 0.661, *T*_max_ = 0.790
10992 measured reflections
2581 independent reflections
1891 reflections with *I* > 2σ(*I*)
*R*_int_ = 0.039

#### Refinement

*R*[*F* ^2^ > 2σ(*F* ^2^)] = 0.037
*wR*(*F* ^2^) = 0.097
*S* = 1.02
2581 reflections
172 parameters
H-atom parameters constrained
Δρ_max_ = 0.31 e Å^−3^
Δρ_min_ = −0.43 e Å^−3^

### 

Data collection: *CrystalClear* (Rigaku, 2005 ▶); cell refinement: *CrystalClear*; data reduction: *CrystalClear*; program(s) used to solve structure: *SHELXS97* (Sheldrick, 2008 ▶); program(s) used to refine structure: *SHELXL97* (Sheldrick, 2008 ▶); molecular graphics: *SHELXTL* (Sheldrick, 2008 ▶); software used to prepare material for publication: *SHELXTL*.

## Supplementary Material

Crystal structure: contains datablocks I, global. DOI: 10.1107/S1600536810006525/hy2283sup1.cif

Structure factors: contains datablocks I. DOI: 10.1107/S1600536810006525/hy2283Isup2.hkl

Additional supplementary materials:  crystallographic information; 3D view; checkCIF report

## Figures and Tables

**Table 1:** Hydrogen-bond geometry (Å, °)

*D*—H⋯*A*	*D*—H	H⋯*A*	*D*⋯*A*	*D*—H⋯*A*
O5—H5⋯O5*W*	0.82	1.82	2.636 (4)	172
O6—H6⋯O4*W*	0.82	1.77	2.587 (4)	172
O1*W*—H1*WA*⋯O3	0.88	2.16	2.956 (3)	151
O1*W*—H1*WA*⋯O6	0.88	2.53	3.081 (3)	122
O1*W*—H1*WB*⋯O6*W*^i^	0.81	1.91	2.698 (3)	165
O2*W*—H2*WA*⋯O2^ii^	0.89	1.96	2.839 (3)	166
O2*W*—H2*WB*⋯O4^iii^	0.82	1.97	2.774 (3)	166
O3*W*—H3*WA*⋯O3^iv^	0.82	2.24	2.869 (2)	134
O4*W*—H4*WA*⋯O2^v^	0.86	1.97	2.829 (3)	177
O5*W*—H5*WA*⋯O3^v^	0.86	2.09	2.927 (3)	166
O6*W*—H6*WA*⋯O1^vi^	0.86	1.92	2.734 (4)	158
O6*W*—H6*WB*⋯O2^vii^	0.84	2.45	3.213 (4)	151

Symmetry codes: (i) 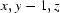; (ii) 
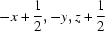
; (iii) 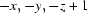; (iv) 
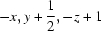
; (v) 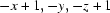; (vi) 
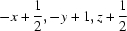
; (vii) 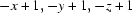.

**Fig. 1.**
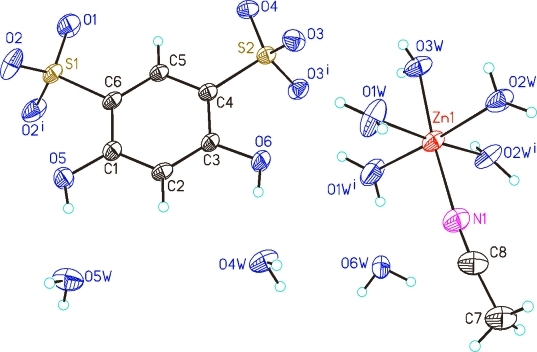
The molecular structure of the title compound. Displacement ellipsoids are drawn at the 30% probability level. [Symmetry code: (i) x, 1/2-y, z.]

**Fig. 2.**
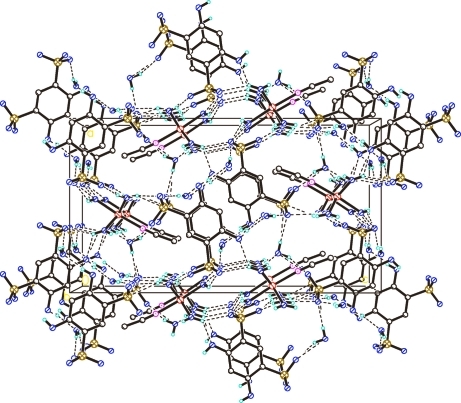
The crystal packing of the title compound viewed along the b axis. H atoms not involved in hydrogen bonding (dashed lines) have been omitted for clarity.

## supplementary crystallographic information

### Comment

Benzenesulfonic acid derivatives have been found wide range of applications in coordination chemistry as ligands, in medicinal chemistry and materials science. There has been an increased interest in the preparation of coordination compounds of benzenesulfonic acid derivatives (Arnold *et al.*, 2001; Du *et al.*, 2006; Junk & Steed, 2007; Xie *et al.*, 2002; Zhang *et al.*, 2009). We report here the crystal structure of the title compound.

The title compound is built up of one [Zn(C_2_H_3_N)(H_2_O)_5_]^2+^ cation, one uncoordinated 4,6-dihydroxybenzene-1,3-disulfonate anion and three uncoordinated water molecules (Fig.1). The distorted octahedral environment around the Zn^II^ ion consists of one acetonitrile ligand and five water molecules. The Zn—O bond distances range from 2.058 (2) to 2.096 (3) Å. The average Zn—O bond distance of 2.078 Å and the Zn—N bond distance of 2.118 (3) Å are similar to the values in other zinc complex (Adarsh *et al.*, 2008; Francis *et al.*, 2003; Lu *et al.*, 2008). The cations, anions and uncoordinated water molecules are linked into a three-dimensional supramolecular network by O—H···O hydrogen bonds (Table 1 and Fig. 2).

### Experimental

Zn(CH_3_CO_2_)_2_ (0.5 mmol) and 4,6-dihydroxybenzene-1,3-disulfonic acid (0.5 mmol) were dissolved in a mixed solution of water (2 ml) and acetonitrile (16 ml). Colorless block crystals of the title compound suitable for X-ray analysis were obtained by evaporation of the solvent in air (yield 63% based on Zn).

### Refinement

H atoms attached to C and O atoms were located in difference Fourier maps and were treated as riding on their parent atoms. The displacement parameters of all H atoms were refined isotropically.

### Crystal data

**Table:**

[Zn(C_2_H_3_N)(H_2_O)_5_](C_6_H_4_O_8_S_2_)·3H_2_O	*F*(000) = 1072
*M**_r_* = 518.80	*D*_x_ = 1.663 Mg m^−^^3^
Orthorhombic, *P**n**m**a*	Mo *K*α radiation, λ = 0.71073 Å
Hall symbol: -P 2ac 2n	Cell parameters from 2210 reflections
*a* = 12.8731 (10) Å	θ = 2.4–27.6°
*b* = 6.9972 (6) Å	µ = 1.46 mm^−^^1^
*c* = 22.9980 (17) Å	*T* = 296 K
*V* = 2071.6 (3) Å^3^	Block, colourless
*Z* = 4	0.32 × 0.24 × 0.16 mm

### Data collection

**Table:**

Rigaku Mercury2 CCD diffractometer	2581 independent reflections
Radiation source: fine-focus sealed tube	1891 reflections with *I* > 2σ(*I*)
graphite	*R*_int_ = 0.039
φ and ω scans	θ_max_ = 27.6°, θ_min_ = 2.4°
Absorption correction: multi-scan (*CrystalClear*; Rigaku, 2005)	*h* = −16→14
*T*_min_ = 0.661, *T*_max_ = 0.790	*k* = −9→9
10992 measured reflections	*l* = −29→29

### Refinement

**Table:**

Refinement on *F*^2^	Secondary atom site location: difference Fourier map
Least-squares matrix: full	Hydrogen site location: inferred from neighbouring sites
*R*[*F*^2^ > 2σ(*F*^2^)] = 0.037	H-atom parameters constrained
*wR*(*F*^2^) = 0.097	*w* = 1/[σ^2^(*F*_o_^2^) + (0.0457*P*)^2^ + 0.7072*P*] where *P* = (*F*_o_^2^ + 2*F*_c_^2^)/3
*S* = 1.02	(Δ/σ)_max_ < 0.001
2581 reflections	Δρ_max_ = 0.31 e Å^−^^3^
172 parameters	Δρ_min_ = −0.43 e Å^−^^3^
0 restraints	Extinction correction: *SHELXL97* (Sheldrick, 2008)
Primary atom site location: structure-invariant direct methods	Extinction coefficient: 0.0021 (5)

### Fractional atomic coordinates and isotropic or equivalent isotropic displacement parameters (Å^2^)

**Table:**

	*x*	*y*	*z*	*U*_iso_*/*U*_eq_	
Zn1	0.05196 (4)	0.2500	0.648181 (17)	0.04642 (16)	
O1W	0.1416 (2)	0.0416 (4)	0.60984 (10)	0.0831 (8)	
H1WB	0.1599	−0.0361	0.6336	0.117 (17)*	
H1WA	0.1627	0.0469	0.5736	0.17 (2)*	
O2W	−0.04689 (16)	0.0412 (3)	0.68120 (8)	0.0615 (6)	
H2WB	−0.0611	−0.0355	0.6555	0.075 (11)*	
H2WA	−0.0580	−0.0096	0.7164	0.122 (16)*	
O3W	−0.0392 (2)	0.2500	0.57265 (12)	0.0579 (8)	
H3WA	−0.0285	0.3485	0.5542	0.15 (2)*	
N1	0.1330 (3)	0.2500	0.72832 (15)	0.0672 (11)	
C7	0.2069 (4)	0.2500	0.8316 (2)	0.0734 (14)	
H7A	0.1782	0.1343	0.8467	0.17 (2)*	
H7B	0.2756	0.2500	0.8350	0.11 (2)*	
C8	0.1698 (4)	0.2500	0.7727 (2)	0.0621 (12)	
S1	0.49094 (8)	0.2500	0.30660 (3)	0.0414 (2)	
S2	0.15787 (7)	0.2500	0.44987 (3)	0.0375 (2)	
O1	0.4012 (2)	0.2500	0.26982 (10)	0.0735 (10)	
O2	0.55305 (17)	0.0800 (3)	0.29897 (8)	0.0669 (6)	
O3	0.13088 (14)	0.0779 (3)	0.48192 (7)	0.0494 (5)	
O4	0.11538 (19)	0.2500	0.39111 (10)	0.0464 (6)	
O5	0.6117 (2)	0.2500	0.41697 (10)	0.0594 (8)	
H5	0.6426	0.2500	0.4482	0.044 (11)*	
O6	0.3153 (2)	0.2500	0.54301 (9)	0.0527 (7)	
H6	0.3605	0.2500	0.5681	0.086 (17)*	
C1	0.5087 (3)	0.2500	0.42656 (13)	0.0394 (8)	
C3	0.3602 (3)	0.2500	0.48999 (13)	0.0359 (8)	
C4	0.2939 (3)	0.2500	0.44165 (13)	0.0351 (8)	
C2	0.4660 (3)	0.2500	0.48202 (13)	0.0407 (9)	
H2	0.5097	0.2500	0.5142	0.047 (10)*	
C5	0.3365 (3)	0.2500	0.38655 (13)	0.0349 (8)	
H5A	0.2929	0.2500	0.3543	0.028 (8)*	
C6	0.4421 (3)	0.2500	0.37869 (13)	0.0357 (8)	
O4W	0.4428 (2)	0.2500	0.62998 (12)	0.0598 (8)	
H4WA	0.4421	0.1514	0.6522	0.108 (16)*	
O5W	0.7285 (2)	0.2500	0.51114 (13)	0.0664 (8)	
H5WA	0.7607	0.1441	0.5160	0.108 (16)*	
O6W	0.2208 (2)	0.7500	0.67248 (12)	0.0559 (7)	
H6WB	0.2849	0.7500	0.6800	0.12 (2)*	
H6WA	0.1983	0.7500	0.7077	0.092 (18)*	

### Atomic displacement parameters (Å^2^)

**Table:**

	*U*^11^	*U*^22^	*U*^33^	*U*^12^	*U*^13^	*U*^23^
Zn1	0.0641 (3)	0.0442 (3)	0.0310 (2)	0.000	−0.00282 (19)	0.000
O1W	0.128 (2)	0.0722 (17)	0.0490 (11)	0.0344 (15)	0.0277 (12)	0.0130 (13)
O2W	0.1018 (17)	0.0502 (12)	0.0326 (9)	−0.0182 (11)	−0.0079 (9)	0.0075 (10)
O3W	0.089 (2)	0.0464 (17)	0.0386 (13)	0.000	−0.0161 (14)	0.000
N1	0.070 (3)	0.085 (3)	0.0461 (19)	0.000	−0.0087 (18)	0.000
C7	0.069 (4)	0.089 (4)	0.063 (3)	0.000	−0.022 (2)	0.000
C8	0.067 (3)	0.053 (3)	0.066 (3)	0.000	−0.018 (2)	0.000
S1	0.0572 (6)	0.0451 (5)	0.0218 (3)	0.000	0.0032 (4)	0.000
S2	0.0406 (5)	0.0411 (5)	0.0307 (4)	0.000	−0.0036 (3)	0.000
O1	0.071 (2)	0.126 (3)	0.0242 (11)	0.000	−0.0051 (12)	0.000
O2	0.1018 (16)	0.0626 (14)	0.0362 (9)	0.0273 (13)	0.0188 (10)	0.0016 (10)
O3	0.0521 (11)	0.0500 (12)	0.0462 (9)	−0.0103 (9)	−0.0010 (8)	0.0091 (9)
O4	0.0487 (15)	0.0545 (17)	0.0360 (12)	0.000	−0.0117 (11)	0.000
O5	0.0416 (16)	0.104 (3)	0.0331 (12)	0.000	0.0003 (11)	0.000
O6	0.0467 (15)	0.087 (2)	0.0244 (10)	0.000	0.0018 (11)	0.000
C1	0.043 (2)	0.045 (2)	0.0302 (15)	0.000	0.0022 (14)	0.000
C3	0.043 (2)	0.042 (2)	0.0227 (13)	0.000	0.0014 (13)	0.000
C4	0.0396 (19)	0.0376 (19)	0.0281 (14)	0.000	−0.0030 (13)	0.000
C2	0.048 (2)	0.051 (2)	0.0232 (14)	0.000	−0.0068 (13)	0.000
C5	0.041 (2)	0.0387 (19)	0.0247 (14)	0.000	−0.0041 (13)	0.000
C6	0.050 (2)	0.0360 (19)	0.0209 (13)	0.000	−0.0003 (13)	0.000
O4W	0.085 (2)	0.0572 (19)	0.0370 (13)	0.000	−0.0126 (13)	0.000
O5W	0.0649 (19)	0.061 (2)	0.073 (2)	0.000	−0.0245 (16)	0.000
O6W	0.0485 (18)	0.071 (2)	0.0479 (15)	0.000	0.0108 (13)	0.000

### Geometric parameters (Å, °)

**Table:**

Zn1—O1W	2.058 (2)	S2—O4	1.458 (2)
Zn1—O2W	2.081 (2)	S2—C4	1.762 (4)
Zn1—O3W	2.096 (3)	O5—C1	1.344 (4)
Zn1—N1	2.118 (3)	O5—H5	0.8206
O1W—H1WB	0.8063	O6—C3	1.349 (4)
O1W—H1WA	0.8769	O6—H6	0.8201
O2W—H2WB	0.8197	C1—C2	1.389 (4)
O2W—H2WA	0.8948	C1—C6	1.395 (4)
O3W—H3WA	0.8204	C3—O6	1.349 (4)
N1—C8	1.126 (5)	C3—C2	1.375 (5)
C7—C8	1.435 (6)	C3—C4	1.402 (4)
C7—H7A	0.9553	C4—C5	1.381 (4)
C7—H7B	0.8878	C2—H2	0.9300
S1—O1	1.432 (3)	C5—C6	1.371 (5)
S1—O2^i^	1.444 (2)	C5—H5A	0.9300
S1—O2	1.444 (2)	O4W—H4WA	0.8585
S1—C6	1.773 (3)	O5W—H5WA	0.8563
S2—O3^i^	1.4541 (19)	O6W—H6WB	0.8433
S2—O3	1.4541 (19)	O6W—H6WA	0.8611
S2—O3	1.4541 (19)		
			
O1W—Zn1—O1W^i^	90.26 (14)	O3^i^—S2—O3	111.83 (16)
O1W—Zn1—O2W^i^	175.32 (9)	O3^i^—S2—O3	111.83 (16)
O1W^i^—Zn1—O2W^i^	90.09 (10)	O3^i^—S2—O4	112.35 (9)
O1W—Zn1—O2W	90.09 (10)	O3—S2—O4	112.35 (9)
O1W^i^—Zn1—O2W	175.32 (9)	O3—S2—O4	112.35 (9)
O2W^i^—Zn1—O2W	89.19 (12)	O3^i^—S2—C4	106.98 (10)
O1W—Zn1—O3W	87.63 (9)	O3—S2—C4	106.98 (10)
O1W^i^—Zn1—O3W	87.63 (9)	O3—S2—C4	106.98 (10)
O2W^i^—Zn1—O3W	87.72 (8)	O4—S2—C4	105.87 (15)
O2W—Zn1—O3W	87.72 (8)	C1—O5—H5	109.6
O1W—Zn1—N1	95.54 (10)	C3—O6—H6	109.5
O1W^i^—Zn1—N1	95.54 (10)	O5—C1—C2	122.7 (3)
O2W^i^—Zn1—N1	89.07 (9)	O5—C1—C6	118.4 (3)
O2W—Zn1—N1	89.07 (9)	C2—C1—C6	118.8 (3)
O3W—Zn1—N1	175.50 (13)	O6—C3—C2	123.0 (3)
Zn1—O1W—H1WB	110.5	O6—C3—C2	123.0 (3)
Zn1—O1W—H1WA	123.4	O6—C3—C4	117.2 (3)
H1WB—O1W—H1WA	125.6	O6—C3—C4	117.2 (3)
Zn1—O2W—H2WB	109.4	C2—C3—C4	119.8 (3)
Zn1—O2W—H2WA	134.9	C5—C4—C3	119.1 (3)
H2WB—O2W—H2WA	110.9	C5—C4—S2	119.6 (2)
Zn1—O3W—H3WA	109.5	C3—C4—S2	121.3 (2)
C8—N1—Zn1	175.3 (4)	C3—C2—C1	120.9 (3)
C8—C7—H7A	102.4	C3—C2—H2	119.5
C8—C7—H7B	114.5	C1—C2—H2	119.5
H7A—C7—H7B	110.7	C6—C5—C4	121.0 (3)
N1—C8—C7	174.6 (6)	C6—C5—H5A	119.5
O1—S1—O2^i^	112.01 (11)	C4—C5—H5A	119.5
O1—S1—O2	112.01 (11)	C5—C6—C1	120.3 (3)
O2^i^—S1—O2	110.9 (2)	C5—C6—S1	118.3 (2)
O1—S1—C6	105.47 (16)	C1—C6—S1	121.4 (3)
O2^i^—S1—C6	108.06 (10)	H6WB—O6W—H6WA	97.8
O2—S1—C6	108.06 (10)		

Symmetry codes: (i) *x*, −*y*+1/2, *z*.

### Hydrogen-bond geometry (Å, °)

**Table:**

*D*—H···*A*	*D*—H	H···*A*	*D*···*A*	*D*—H···*A*
O5—H5···O5W	0.82	1.82	2.636 (4)	172
O6—H6···O4W	0.82	1.77	2.587 (4)	172
O1W—H1WA···O3	0.88	2.16	2.956 (3)	151
O1W—H1WA···O6	0.88	2.53	3.081 (3)	122
O1W—H1WB···O6W^ii^	0.81	1.91	2.698 (3)	165
O2W—H2WA···O2^iii^	0.89	1.96	2.839 (3)	166
O2W—H2WB···O4^iv^	0.82	1.97	2.774 (3)	166
O3W—H3WA···O3^v^	0.82	2.24	2.869 (2)	134
O4W—H4WA···O2^vi^	0.86	1.97	2.829 (3)	177
O5W—H5WA···O3^vi^	0.86	2.09	2.927 (3)	166
O6W—H6WA···O1^vii^	0.86	1.92	2.734 (4)	158
O6W—H6WB···O2^viii^	0.84	2.45	3.213 (4)	151

Symmetry codes:  (ii) *x*, *y*−1, *z*; (iii) −*x*+1/2, −*y*, *z*+1/2; (iv) −*x*, −*y*, −*z*+1; (v) −*x*, *y*+1/2, −*z*+1; (vi) −*x*+1, −*y*, −*z*+1; (vii) −*x*+1/2, −*y*+1, *z*+1/2; (viii) −*x*+1, −*y*+1, −*z*+1.
